# Decision Making for Healthcare Resource Allocation: Joint v. Separate Decisions on Interacting Interventions

**DOI:** 10.1177/0272989X18758018

**Published:** 2018-04-23

**Authors:** Helen Dakin, Alastair Gray

**Affiliations:** Nuffield Department of Population Health, University of Oxford, UK; Nuffield Department of Population Health, University of Oxford, UK

**Keywords:** cost-effectiveness, economic evaluation, healthcare decision making, health technology assessment, interactions

## Abstract

Standard guidance for allocating healthcare resources based on cost-effectiveness recommends using different decision rules for independent and mutually exclusive alternatives, although there is some confusion around the definition of “mutually exclusive.” This paper reviews the definitions used in the literature and shows that interactions (i.e., non-additive effects, whereby the effect of giving 2 interventions simultaneously does not equal the sum of their individual effects) are the defining feature of mutually exclusive alternatives: treatments cannot be considered independent if the costs and/or benefits of one treatment are affected by the other treatment. The paper then identifies and categorizes the situations in which interventions are likely to have non-additive effects, including interventions targeting the same goal or clinical event, or life-saving interventions given to overlapping populations. We demonstrate that making separate decisions on interventions that have non-additive effects can prevent us from maximizing health gained from the healthcare budget. In contrast, treating combinations of independent options as though they were “mutually exclusive” makes the analysis more complicated but does not affect the conclusions. Although interactions are considered by the World Health Organization, other decision makers, such as the National Institute for Health and Care Excellence (NICE), currently make independent decisions on treatments likely to have non-additive effects. We propose a framework by which interactions could be considered when selecting, prioritizing, and appraising healthcare technologies to ensure efficient, evidence-based decision making.

Two different decision rules are used to interpret economic evaluations and allocate resources between multiple competing uses,^1-13^ depending on whether interventions are independent or mutually exclusive.

When the alternatives are considered to be independent, the decision rule is simply to compare the incremental cost-effectiveness ratios (ICERs) for the different interventions (each relative to their next best non-dominated alternative) side by side.^2-10,14^ If we have a league table showing the cost-effectiveness and budget impact of all possible independent healthcare interventions, we can maximize the amount of health gained by adopting interventions in ascending order of ICER until the available healthcare budget is exhausted. Alternatively, if we have an estimate of the shadow price of a quality-adjusted life year (QALY), we can simply adopt all interventions with ICERs below this ceiling ratio.

Conversely, only one intervention from a set of mutually exclusive alternatives will be adopted at a time. In this situation, the decision rule requires calculating the ICER for each option compared with its next best non-dominated alternative.^1-3,6,7,9-11,13^ We then identify the cost-effectiveness frontier^2,3^ by excluding any interventions that are strongly or weakly dominated by others and adopt the most effective intervention that lies on the cost-effectiveness frontier and has an ICER below our ceiling ratio.^6,10,13^ Using net monetary benefit (NMB), or net health benefit, simplifies the procedure further: we can simply calculate the NMB of all alternatives at the appropriate ceiling ratio and adopt the intervention with highest NMB.^1,2,11,12^

However, the distinction between independent and mutually exclusive interventions is not always clear. This paper examines the role of interactions in determining when each decision rule should be used. We define interactions as situations where the absolute incremental costs and health benefits of one intervention are affected by whether another intervention is given. We argue that interactions are more pervasive than commonly thought, and need to be considered carefully within economic evaluation and health technology assessment (HTA).

The next section reviews published definitions of “independent” and “mutually exclusive.” We then describe situations in which interactions are likely to arise and use numerical examples and published mathematical proofs to assess the impact of using the “wrong” decision rule: i.e., making separate decisions on interventions that interact, and treating combinations of independent options as though they were mutually exclusive. The paper concludes with a discussion of how HTA organizations and decision makers, such as the National Institute of Health and Care Excellence (NICE) and the World Health Organization (WHO), currently deal with interactions, and proposes a framework for identifying and accounting for interactions within economic evaluations and HTA that could enable more efficient use of healthcare resources.

## Definition of Mutually Exclusive and Independent Alternatives

We conducted a literature review of published definitions of “independent” and “mutually exclusive” in the context of healthcare decision making.^i^ The review identified substantial variation among the 14 published definitions (Appendix 1). Thirteen texts defined “mutually exclusive” literally, stating that interventions are mutually exclusive if patients cannot receive both. However, there are relatively few interventions that are impossible to implement together (rare examples include different levels of the same intervention or different approaches to irreversible surgery). Seven definitions stated that all interventions given to the same patient population are mutually exclusive and/or that interventions given to different populations are independent. However, 9 of the published definitions suggested that the defining feature that determines whether interventions are mutually exclusive or independent is whether implementing one intervention affects the costs or effectiveness of the other: in other words, whether the treatments interact, rather than having additive effects (whereby the effect of A+B is equal to the effect of A plus the effect of B).^ii^

These 9 definitions suggest that whenever interventions (e.g., A and B) interact (i.e., have non-additive effects), we should treat the combinations of interrelated interventions (e.g., no intervention, A, B and A+B) as mutually exclusive options, comparing the intervention combinations incrementally and selecting the single strategy that maximizes NMB for that population. Conversely, the definitions imply that, if there is no interaction between interventions (i.e., additive effects), we should treat A and B as independent options and compare their ICERs against the ceiling ratio separately, adopting those interventions with ICERs below our ceiling ratio.

However, only the 2 WHO definitions^18,19^ mention the word “interaction,” and use slightly different terminology. They define mutually exclusive interventions as those that cannot be implemented together and distinguish them from interventions that can be given together but interact, and from independent interventions that do not interact. They recommend that combinations of interacting interventions are considered as a “cluster” of interdependent interventions and evaluated incrementally like mutually exclusive alternatives.

## Situations When Interactions Are Likely to Arise

Based on published studies and economic evaluations that we have been involved in, we developed 13 worked examples based on real or hypothetical data to illustrate mechanisms by which interventions may have non-additive effects on costs, QALYs, or NMB (Appendix 2). We grouped these mechanisms into 5 categories (Table 1), which could each arise with any type of healthcare intervention.

**Table 1:** A Taxonomy of Types of Interactions^a^

Interactions between interventions given to the same patients	1: Direct pharmacological, behavioral or biological mechanisms	
	2: Scale effects	2a: Multiplicative effects on the risk/hazard/odds of clinical events or mortality
		2b: Multiplicative effects between quality and length of life
		2c: Multiplicative effects on cost
		2d: Multiplicative effects between immediate mortality and remaining life expectancy
		2e: Non-multiplicative scale effects
	3: Non-additive marginal effects on HRQoL	3a: Diminishing marginal effects on HRQoL
		3b: Increasing marginal effects on HRQoL
		3c: Ceiling effects on quality and/or length of life
	4: Patient pathway	4a: Earlier intervention affects costs, or benefits of later intervention (or vice versa)
		4b: Interactions between diseases
		4c: Effect of comorbid conditions on treatment costs
		4d: Future costs: i.e., healthcare resource use in years of life gained
5: Interactions between interventions given to different patients treated by the same staff or in the same healthcare facilities		

HRQoL, health-related quality of life.

a Worked examples of each type of interaction are shown in Appendix 2.

First, pharmacological, behavioral, and biological mechanisms can introduce interactions^20–22^ (category 1). For example: one drug may reduce or accelerate the metabolism of the other (potentially influencing efficacy or causing adverse effects); giving a second intervention may affect compliance with the first intervention;^23^ or biological mechanisms may mean that adding A in patients already receiving B has less impact than when A is given alone.

Second, interactions can arise from scale effects^20,24–27^ (category 2). Interventions generally have approximately multiplicative (i.e., proportional) effects on the risk of clinical events (e.g., death or stroke),^21,28^ increasing or decreasing risk, odds, or hazard by a certain percentage rather than an absolute amount. This means that the absolute effect of treatment is smaller for low-risk patients^29^ and therefore smaller for patients who are already receiving treatment. If clinical events increase costs and reduce quality or length of life, interventions (e.g., statins and antihypertensives) that reduce event rates by X% will have synergistic interactions for costs and antagonistic interactions for QALYs (category 2a).^17,iii^ All life-extending interventions are likely to have multiplicative/proportional effects on all-cause mortality, even if they are used to treat different diseases in the same patients. Synergistic interactions for QALYs can arise when one intervention (e.g., smoking cessation) extends life expectancy, thereby increasing the QALY gains from another intervention (e.g., joint replacement) improving health-related quality of life (HRQoL), and vice versa (category 2b). Many interventions and covariates also have multiplicative effects on cost^30^ (category 2c); e.g., interventions may halve the length of stay or numbers of consultations, generating greater savings for high-cost patients receiving another treatment. Large, synergistic interactions for costs have also been observed within studies comparing dosing regimens of different drugs.^31^

Third, giving further healthcare interventions to the same patients may yield diminishing marginal returns for utilities: Because comorbid conditions generally have non-additive effects on utility,^32,33^ the improvement in HRQoL from receiving 2 equally effective interventions may be less than double that from receiving one intervention, leading to an antagonistic interaction (category 3). These effects are built into several utility measures. The Health Utilities Index (HUI) assumes that attributes have a multiplicative effect on utility,^33^ whereas the UK EQ-5D-3L tariff can introduce antagonistic (category 3a) or synergistic (category 3b) interactions. Ceiling effects can also produce diminishing marginal returns, because no combination of interventions can improve utilities above 1, and patients cannot accrue more than 5 life-years within a 5-year trial (category 3c).

Interactions can also arise from the patient pathway (category 4). For example, the costs and benefits of screening will always depend on the prevalence of the condition (which, in turn, depends on the preventative measures adopted) and on what interventions are used to diagnose and treat the cases identified^34^ (category 4a). Similarly, the costs and benefits of preventative interventions will depend on the costs and effectiveness of subsequent screening and treatment: e.g., prevention may have little value if all cases are diagnosed promptly and receive a cheap, highly effective cure. Codependent technologies (e.g., trastuzumab and human epidermal growth receptor-2 [HER2] testing^35^) represent an extreme form of interaction: a test to determine the suitability of treatment has no benefit if patients will not subsequently be treated, whereas the test increases the health benefits from treatment and/or reduces costs. The costs, benefits, and range of options for second-line therapy may also be affected by what intervention was given first-line (and vice versa), particularly if drug resistance or toxicity develop during treatment.

Interactions between interventions can also arise from interactions between diseases^36^ (category 4b). For example, if heart failure changes the risk, case-fatality, cost, or HRQoL of stroke,^36^ interventions reducing the risk of heart failure will also indirectly affect the cost-effectiveness of interventions to prevent and/or treat stroke, even if they do not influence the incidence of stroke in patients without comorbid heart failure. The cost of delivering interventions may also be affected by how comorbid conditions (e.g., obesity) are managed (category 4c). Introducing new interventions that increase the cost of treatment for any common condition will also increase the cost accrued in the years of life gained from any life-saving intervention if such “future costs”^37,38^ are included in the analysis (category 4d).

Although the above types of interactions arise between interventions given to the same patients, interactions are also possible between interventions given to different patients within the same healthcare system (category 5). Equipment purchased for one intervention may also be used for other patients, affecting their costs and health gains. Interventions reducing length of stay may increase the length of stay or staffing ratios for other interventions, which will introduce interactions for cost and, potentially, health effects.

Complex interventions and changes to service delivery/organization will also change the costs and benefits of numerous interventions; e.g., setting up or reorganizing a primary care service to offer measles vaccination may change the feasibility, cost and outcomes of tuberculosis treatment that is then delivered in the same center. Similarly, whole-genome sequencing for one condition (e.g., cystic fibrosis) may, incidentally, detect other mutations (e.g., BRCA1), which may affect the costs and benefits of screening and treatment of other conditions in the whole family.^39^ Conversely, adding an additional intervention into a healthcare service can affect compliance or waiting times^40^ for other interventions and introduce interactions between the new service and existing ones delivered by the same healthcare professionals.

However, with the exception of complex interventions, large interactions between interventions given to different patients are likely to occur less commonly than interactions between interventions for the same patients. This may explain the apparent contradiction between the definitions described above: because economically important interactions are substantially more likely between interventions given to the same patients than interventions given to different patients (at least for “simple interventions”), “patient group” is a useful rule of thumb to identify situations with potentially important interactions.

## Implications of Using the “Wrong”Decision Rule

Having shown that interactions may arise in many circumstances, and that their presence determines which decision making rule is recommended, we now use a numerical example and published mathematical proofs to evaluate the implications of using the “wrong” decision rule: i.e., making separate decisions about technologies that interact, and making a joint decision between different combinations of technologies that are truly independent.

Statins and antihypertensives have non-additive effects on costs and QALYs, as both reduce the rate of cardiovascular events by a certain percentage (see Appendix 2, Example 2a). If we were to make a joint decision on statins and antihypertensives allowing for this interaction, we would evaluate no treatment, antihypertensive only, statin only, and statin + antihypertensive incrementally as 4 mutually exclusive treatment combinations (see Appendix 2, Table A5). This incremental analysis suggests that the most cost-effective treatment at a £20,000/QALY ceiling ratio is statin monotherapy, which costs £13,541/QALY v. no treatment, whereas combination therapy costs £54,760/QALY v. statin monotherapy.

In contrast, making separate decisions on statins and antihypertensives (as was done by NICE^41,42^) and ignoring interactions between them would lead to us adopting both statin (£13,541/QALY v. no statin) and antihypertensive (£16,119/QALYs v. no antihypertensive; see Appendix 2, Table A6). We therefore implement combination therapy even though this does not have the highest NMB when we allow for interactions.

The literature on factorial trials demonstrates that ignoring interactions and estimating the average treatment effects for A across patients with and without B gives a biased estimate of the effect of A alone v. no treatment, unless the true interaction is equal to zero.^15,20,43^ Even if we estimate the effect of A v. no A and B v. no B only in patients who received no other treatment, we will get a biased estimate of the effect of A+B together unless the interaction equals zero.^iv^ Conducting separate economic evaluations on A and B also means that we cannot compare treatment combinations incrementally, excluding dominated alternatives. Consequently, analyses ignoring interactions may give biased estimates of incremental effectiveness, costs, and NMB whenever the interaction is not equal to 0.

Accounting for interactions will change our decision about which treatment to adopt whenever there is a qualitative interaction that changes which treatment has highest NMB.^17^ If we make decisions on many sets of interacting interventions, we are therefore likely to adopt a different set of interventions if we make separate decisions (ignoring interactions), compared with the interventions that would be adopted if we make joint decisions (considering all interactions). Laska et al.^11^ demonstrated that the standard decision rules for independent and mutually exclusive interventions produce more effectiveness from the fixed budget than any other possible allocation of resources. Because these decision rules rely upon accurate estimates of incremental cost, incremental effectiveness, and ICERs, any resource allocation based on biased estimates of these parameters must produce less health from the fixed budget than a resource allocation based on unbiased estimates.

In contrast, when there is no interaction between 2 interventions, we will make the same resource allocation decision regardless of whether we make a joint or separate decision,^17^ because incremental costs and effectiveness are unbiased^15,20,43^ and not affected by the other intervention. For example, we make the same resource allocation decisions and obtain the same ICERs regardless of whether we make joint or separate decisions on independent treatments for ovarian cancer and benign prostatic hypertrophy (see Appendix 2, Tables A31-A32).

Furthermore, interactions will only change treatment adoption decisions if they affect which treatment has highest NMB,^17^ which can only arise if the interaction is qualitative: i.e., is larger than the incremental NMB and has the opposite sign. Such a qualitative interaction for NMB occurs within Example 2a, which means that the analysis treating statins and antihypertensives as independent options gives the misleading conclusion that antihypertensive + statin is best value for money, when in fact statin alone has highest NMB. Interactions are therefore unlikely to change the conclusions if they are small, or if the intervention will be cost-effective (or never be cost-effective) regardless of the size of interactions.

## Current Decision Making Methods

HTA organizations such as NICE, Scottish Medicines Consortium (SMC), Pharmaceutical Benefits Advisory Committee (PBAC), and the Canadian Agency for Drugs and Technologies in Health (CADTH) currently make separate resource allocation decisions on different interventions used for the same conditions. For example, NICE made separate recommendations for cervical cancer screening (TA69) and cervical cancer treatment (TA183), which did not explicitly discuss the interactions between them. The decision about whether to allow for interactions between interventions is left up to individual analysts, with no explicit guidance given in the “scope” document, in which NICE defines the decision problem.

Making separate decisions on different interventions simplifies decision making and enables decisions to be made relatively quickly by different teams. However, this approach has several limitations. First, considering interventions one at a time means that the study question does not explicitly address what, if any, concomitant interventions given alongside treatment and means that interactions may be ignored, leading to suboptimal decision making. Second, even in situations where A+B combination therapy is inappropriate, evaluating interventions one at a time can mean that A is not considered as a comparator when B is evaluated, which may mean that dominance is overlooked and that ICERs are calculated relative to a comparator that does not lie on the cost-effectiveness frontier.^v^ Third, recommendations based on separate decisions frequently do not explicitly state whether interventions are recommended alone or in combination with other interventions. For example, NICE commonly recommends interventions “as an option for” the condition in question, without explicitly discussing what, if any, concomitant intervention is assumed or recommended.

By contrast, the WHO framework for generalized cost-effectiveness analysis (GCEA) models clusters interrelated interventions (e.g., cervical cancer prevention, screening and treatment^47^) simultaneously, and explicitly allows for interactions between interventions.^18,48^ Different combinations of interventions are compared against a “null” (comprising costs and health benefits if the entire cluster of interventions was withdrawn) to assess the cost-effectiveness of current interventions and ensure that results can be generalized to other populations. The WHO population model (PopMod) also directly allows for different types of interactions between diseases.^36^ However, the evidence on interactions is frequently weak and relies on assumptions of multiplicative effects.^18,49^

## A Framework for Identifying Interactions

This paper has shown that it is always correct to evaluate interventions jointly while making separate decisions on interacting interventions (even if there is no interaction), and that failing to consider interactions will often fail to maximize health gains from the budget.

In practice, small interactions are likely to arise between nearly all interventions given to the same patients and many of those used in different patients in the same healthcare system. However, considering different combinations of multiple interventions incrementally in a joint decision would greatly increase the complexity, cost, and length of HTA processes, and raise practical challenges. Accurate, efficient HTA therefore requires us to identify which interactions must be considered and which can safely be ignored.

**Figure 1:**
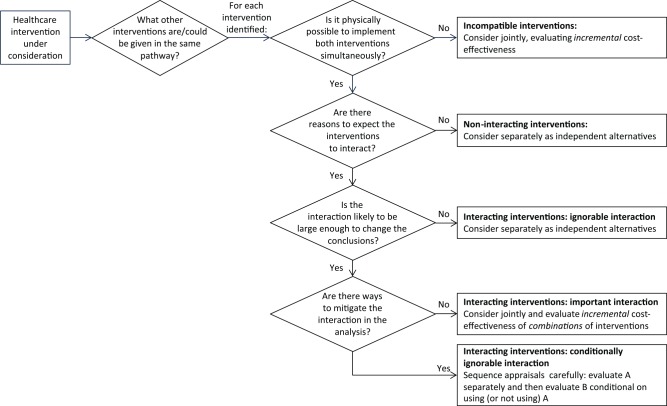
Flow diagram illustrating proposed terminology and decision rules.

Figure 1 presents a framework that could be used to identify potential interactions, allowing for those interactions likely to change the conclusions of the analysis while ignoring small, unimportant interactions. Appendix 3 presents a tabular version of the framework with a worked example.

When assessing the impact of interactions, we must first identify what other interventions are (or could be) given in the same treatment pathway. This may include: interventions targeting other conditions (or even other patients), particularly complex interventions; conditions where many patients have a comorbid condition; or conditions where the intervention requires purchase of costly equipment/resources likely to be used for other conditions.

For each intervention identified, we should then assess whether it is possible to implement it at the same time as the intervention of interest. Depending on the decision problem, this question may be posed at the level of individual patients (e.g., is it possible to give both interventions to the same patient?) or at the level of healthcare systems (e.g., is it possible to implement both interventions within the same hospital/region?). We can then use the taxonomy in Table 1 to assess whether the interventions interact; i.e., whether one intervention is likely to affect the incremental costs and/or health outcomes of the other.

Next, we must consider whether any interaction is likely to be large enough to change the conclusions. Interactions likely to be much smaller than the differences between treatments can generally be ignored. Furthermore, interactions are unlikely to change the conclusions if both interventions will be highly cost-effective (or will never be cost-effective) regardless of the interactions. Whereas all interventions given to overlapping populations are likely to interact to some extent (e.g., through impacts on mortality), such interactions may frequently be small and unlikely to change the conclusions of the analysis. This is particularly so if interventions have only a small impact on total life expectancy or if the overlap between populations is small. It may therefore be appropriate to make a pragmatic decision about whether the added complexity of making a joint decision is justified, given the strength of the interactions.^18^

Some interventions may also interact in the long term but be independent in the short term. For example, the cost-effectiveness of screening for cervical cancer will depend on the availability and coverage of human papillomavirus vaccination, which will reduce the incidence, whereas the cost-effectiveness of vaccination will depend on the screening conducted in the future.^47^ However given the delay between vaccination at age 12 to 13, and screening from age 25, it may be reasonable to make separate decisions about vaccination and screening today, providing that these are reviewed regularly to allow for changes in future incidence and intervention patterns.

The terms “independent” and “mutually exclusive” are currently used inconsistently in the literature, potentially causing confusion. We propose using the terms “incompatible” (i.e., the technologies cannot be used together) and “interacting” (i.e., the technologies can be used together but have non-additive effects on costs and/or QALYs) in place of “mutually exclusive.” If interactions are nonexistent or ignorable, separate decisions can be made, treating the options as independent. If the interventions are incompatible or have important interactions, it will generally be necessary to make a joint decision and evaluate incremental cost-effectiveness.

However, the effect of some interactions can be mitigated by careful sequencing of appraisals. For example, the options for cervical cancer treatment could be appraised before assessing screening/prevention. Although screening may increase the chance that cancers are detected at an early stage, providing that costs, HRQoL and mortality within each stage are independent of screening, we can make stratified decisions about the appropriate treatment for any given stage, independent of what screening is offered. The appraisal of screening or prevention strategies could be started after the treatment appraisal was completed and could assume that we have already adopted the most cost-effective strategy for treating the cancers detected (example 4a). It may also be appropriate to schedule appraisals that evaluate first-line treatment after appraisals evaluating second or subsequent-line treatment, so that the pathway of subsequent treatment can be optimized before first-line treatment is evaluated. Using this strategy, we can make optimal decisions by first comparing all possible last-line therapies in a range of different subgroups who have already failed to respond to/tolerate different sets of treatments previously. Once the last-line treatment is optimized for each group, we can evaluate the penultimate-line treatment, assuming that everybody has the optimal last-line treatment (i.e., assuming technical efficiency). Each line of treatment can be evaluated in reverse order, eventually enabling us to assess first-line treatment conditional on patients subsequently receiving the optimal sequence of treatments in their subsequent care. This approach simplifies the decision about first-line treatment compared with the alternative, namely, modeling all possible sequences of drugs to identify the best treatment pathway overall, which can require comparisons between hundreds of different treatment strategies (e.g., Dakin et al^46^).

If intervention A is likely to be highly cost-effective (or extremely poor value for money) regardless of whether interactions are considered, whereas the cost-effectiveness of B is likely to depend on A, we can ignore interactions when evaluating A, and subsequently assess B based on the assumption that A has already been adopted (or will not be adopted).

Even after ignoring unimportant interactions and sequencing decisions logically, many clusters of interventions will still need to be evaluated jointly. For example, prevention and screening/treatment should be considered jointly when there is only a short delay before onset of disease (e.g., vaccination and prophylactic treatment for influenza). Similarly, decisions about dosing regimen cannot be made separately from decisions about which treatment to give and it would be meaningless to evaluate codependent technologies separately.

Joint decisions on interacting treatments will require evidence or assumptions on the magnitude and direction of interactions. Adequately powered factorial randomized controlled trials would provide the best evidence on interactions.^21^ Even factorial trials lacking prospective collection of resource use or HRQoL data can inform the interactions used in decision-analytical models. Factorial trials with economic evaluations can also directly assess the magnitude of interactions for costs, QALYs, and NMB, and evaluate the cost-effectiveness of both interventions simultaneously, considering any observed interaction.^17^

Information on interactions can also be provided by network meta-analysis, which synthesizes evidence on an entire network of interventions and uses head-to-head randomized controlled trials and indirect comparisons to estimate the relative efficacy of each intervention compared with all alternatives.^50–52^ However, more research is needed on the best ways to estimate interactions and allow for factorial trials within network meta-analyses.

In principle, subgroup analyses stratifying patients by concomitant treatment could be used to estimate interactions or test whether effects are additive or multiplicative. However, subgroup analyses will not give unbiased estimates of the efficacy of any intervention to which patients were not randomly assigned. (For example, a trial randomizing patients to receive A or placebo that stratified patients into those who received concomitant B and those who did not, will give unbiased estimates of the efficacy of A v. not-A with/without B and could be used to inform decisions about which patients should receive A. However, this study would not inform a joint decision about whether patients should receive A, B, or A+B, since selection bias could confound any differences between the groups with and without B.)

Factorial trials, subgroup analyses, or epidemiological studies may also provide evidence suggesting that interventions have multiplicative effects on outcomes, such as reducing the rate, odds or probability of clinical events by a certain percentage. Even in the absence of such studies, it may be reasonable to assume, as is commonly done in WHO-CHOICE studies,^18,49^ that interventions affecting the chance of subsequent events have multiplicative effects.

Interactions that arise from the clinical pathway can also be built into the model structure based on expert opinion or guidelines. For example, models assessing the cost-effectiveness of screening can allow for different downstream treatment options, whereas those on first-line treatment can allow for second-line (and subsequent) treatments. Expert opinion or pilot studies could be used to evaluate the impact of changes to service organization or delivery on the costs and benefits of the interventions delivered through that service.

In many cases, the evidence on interactions may be weak. However, given that decisions cannot be deferred, it is more appropriate to use the most plausible assumptions about interactions within any model, rather than assuming that the interaction is zero. Uncertainty around interactions should be explored in sensitivity analyses alongside other forms of model uncertainty. Furthermore, making a joint decision between mutually exclusive combinations forces analysts and decision makers to explicitly consider the direction and magnitude of interactions, prompts the collection of evidence on interactions, and enables the explicit consideration of the uncertainty around the interaction.

## Conclusions

We have shown that interactions determine whether it is appropriate to make independent decisions on different interventions, and have developed a taxonomy of mechanisms by which interactions may arise. We demonstrated that making a joint decision on multiple interventions, considering interactions, will always maximize health gains from the budget, whereas making independent decisions on interacting technologies can lead to inefficient resource allocation decisions.

HTA organizations, such as NICE, could improve decision making by using our framework to consider the likelihood, type, and magnitude of interactions among interventions at all stages in the appraisal process and allow for potentially influential interactions in decision making. Using Figure 1 at the pre-scoping or horizon-scanning stage could help sequence appraisals to mitigate interactions and identify which interventions are likely to have no important interactions and can be evaluated separately v. which must be considered jointly (e.g., as guidelines or multiple technology appraisals). Manufacturers and academic groups could be advised to consider the interactions identified at the scoping stage and address the most appropriate research questions. At present, this is left to the analysts’ discretion. Well-conducted analyses following existing best-practice guidelines may implicitly account for some interactions, although other studies may benefit from explicitly considering the potential for interactions between interventions. Other researchers may also use the framework to plan economic evaluations and ensure that the assumptions they make about concomitant interventions and interactions between interventions are explicitly stated in publications.

## Supplementary Material

Appendix_1

Appendix_2

Appendix_3

## Notes

i. The literature review was conducted by one author (HD). Definitions were identified by searching Google scholar (first 100 hits only) and Medline (through PubMed) on 5 November 2013 and 15 February 2016 for terms including ‘‘mutually-exclusive’ cost-effective,’ ‘independent option cost-effective’ and ‘independent alternative cost-effective.’ Key health economic textbooks and the reference lists from identified papers and books were also reviewed. Fourteen references with definitions were identified (Appendix 1)

ii. We follow the factorial trial literature in defining interactions as the effect of interventions A and B together (compared with no intervention), minus the sum of the effects of interventions A and B separately.^15,16^ In each case, treatment effects are defined as the difference in any outcome (whether that is costs, QALYs or other endpoints) between the intervention in question and no intervention. Although treatment effects can be analyzed on a logarithmic scale or subjected to other transformations, we focus on treatment effects measured on a natural scale and therefore define interactions as deviations from additive effects, because efficient allocation of healthcare resources must be based on absolute differences in costs and QALYs measured on a natural scale.^17^

iii. Interactions may be either *antagonistic* (i.e., the effect of A+B together is less than the effect A and B individually) or *synergistic* (i.e., the effect of A+B is greater than the effect of A and B individually). Very large antagonistic interactions may be *qualitative* if adding A to B has the opposite effect to adding A to no treatment, which means that the interaction changes the ranking of treatments with respect to the outcome in question.

iv. Interactions mean that the effect of adding A to B ($\mu_{ab}-\mu_{b}$) is not equal to the effect of adding A to no treatment ($\mu_{a}-\mu_{o}$). Consequently, adding the effect of A alone ($\mu_{a}-\mu_{o}$) and the effect of B alone ($\mu_{b}-\mu_{o}$) to the outcomes with no treatment ($\mu_{o}$) will only equal the outcomes for A+B if there is no interaction. $\mu_{ab}=\mu_{0}+(\mu_{b}-\mu_{0})+(\mu_{a}-\mu_{0})$ only if the interaction^15,16^ ($\mu_{0}+\mu_{ab}-\mu_{a}-\mu_{b}$) equals zero. Making separate decisions based on the effect of A alone ($\mu_{a}-\mu_{o}$) and the effect of B alone ($\mu_{b}-\mu_{o}$) therefore gives biased estimates of the effect of $\mu_{ab}$ whenever the interaction does not equal zero but gives unbiased estimates if the interaction equals zero

v. For example, NICE evaluated tenofovir and entecavir in separate appraisals and recommended both treatments for hepatitis B,^44,45^ despite entecavir being substantially more expensive, with no evidence of higher efficacy.^46^
