# Supplementary material for: Decision Making for Healthcare Resource Allocation: Joint v. Separate Decisions on Interacting Interventions
Source: Med Decis Making. 2018 Apr 23;38(4):476–86. doi: 10.1177/0272989X18758018 (PMC5949981; doi:10.1177/0272989X18758018)
Supplement: Appendix_1 [file Appendix_1.pdf]

Appendix 1: Published definitions of "mutually exclusive" and "independent"

Table A1. Published definitions of "mutually exclusive" and "independent", categorised into: **L**, Literal definition (interventions are considered mutually exclusive if patients cannot receive both); **P**, Interventions for the same patient group are considered mutually exclusive; **I**, Interventions are considered mutually exclusive if the costs or effects depend on the other intervention, although the word "interaction" is not mentioned; **I+**, Interactions are mentioned as a defining feature of mutually exclusive interventions; **I-**, Definition states that independent interventions may interact.

Publication	Definition of "mutually exclusive"	Definition of "independent"	Type of definition
Briggs, 2000[53]	"Mutually exclusive programmes involve the same group of patients and, therefore, one or the other must be chosen. Patients can be screened yearly or biennially, but not both. Similarly, patients can receive either a 40 or 80mg regimen for a particular drug."	"Independent programmes can be implemented either singly or jointly, so in the case of the ICD [implantable cardioverter defibrillator], the device could be implanted into patients with a high ejection fraction or those with a low ejection fraction, or into both types of patient. Such a decision should be based on the incremental cost effectiveness of the ICD for these 2 types of patients compared with alternative (medical) management."	L P
Drummond et al., 2015[1]	"Either/or decisions" (page 98)	"For example, choosing which interventions should be offered to different subgroups of patients with the same condition are not mutually exclusive choices." (Page 103)	L
Drummond et al., 2005[54]*	"The various alternatives within each programme are assumed to be mutually exclusive, in that if a patient receives one of the treatments in each programme, they will not receive the others" (pages 127-130)	"The treatment used in one patient group is assumed to be independent of the treatments used in other groups. That is, the costs and health effects of a treatment in one patient group are not affected by the treatment alternative chosen in any other patient group." (pages 127-30)<br>"The costs and health effects in one patient group are not affected by the treatment alternative in any other patient group" (page 12)	L I P
Elbasha and Messonnier, 2004[55]	"Programs in a cluster are said to be locally mutually exclusive if an individual receives a service of one program, and he/she cannot obtain the service of another program from the same cluster [22]. Examples of locally mutually exclusive programs are cancer detection programs defined by the frequency of screening. An individual cannot be screened both annually and biennially. However, it is assumed that two individuals can receive the services of two different programs from the same cluster. For example, one individual is annually screened, whereas the other is screened every 2 years. That is, programs are not globally mutually exclusive."		L

Publication	Definition of "mutually exclusive"	Definition of "independent"	Type of definition
Evans et al., 2005[19]	"If one is chosen, another cannot be."<br>"Mutually exclusive interventions must replace an existing intervention"<br>*However, the authors do recommend evaluating all combinations of interventions allowing for interactions*	"Can be done at the same time in a population, with or without interactions."<br>"Can be added to existing interventions"	L I-
Gray et al., 2011[2]	"Clearly, it would be possible to implement both primary *and* secondary prevention; these are independent policies affecting different patient groups, and so the appropriate approach would not be to calculate the incremental cost-effectiveness of primary versus secondary prevention, but rather to calculate the incremental cost-effectiveness of statins in each relative to the next best alternative for that group of patients. The resulting ICERs could then be compared to see which offered the best value for money. Similarly, policies aimed at different age groups are independent, and a direct incremental calculation of the cost-effectiveness in one age group versus another would not be appropriate. However, different doses of statin are a different matter. Giving a 20 mg/day dose cannot be considered independently of giving the same person a 40 mg/day or 80 mg/day dose; these are mutually exclusive alternatives." (page 15)		L P
Heshmat, 2001[56]	"…Two programs, A and B, are mutually exclusive if implementing program A (B) means that program B (A) cannot also be implemented. Mutually exclusive programmes can be viewed as programmes for the same population (e.g. two alternative drugs for ulcer patients)."	"Two programs, A and B, are defined as independent if the costs and effectiveness of programme A (B) are not affected by whether program B (A) is implemented or not. The two programmes are viewed as applying to two different populations; an example being the treatment of ulcer patients and the treatment of arthritis patients...."	L I P
Hunink et al., 2001[3]	"In many health applications of CEA, however, the choices involve two or more interventions that, by definition, cannot be selected at the same time – not just a yes/no choice for each alternative. The choice among four strategies for breast cancer screening, each involving a different screening interval, is such a choice among mutually exclusive, or *competing choices*. Another is the choice between two different surgical procedures. This situation contrasts with the choice facing a public health agency of whether to implement an anti-smoking campaign and/or a skin cancer screening programme, since the decision to perform one service does not inherently preclude a decision to perform the other one […] The basic difference between these situations is that in competing choice situations such as that among breast cancer screening methods, the alternatives are not independent. In other words, the choice of the first alternative influences the benefit to be gained (or the cost incurred) by the second. In a noncompeting situation – such as the public health agency with a financial budget or the clinician with a time budget – the benefits of each programme can be added. In the competing choice situation, they cannot." (page 277)<br>"If you select Program M1 you cannot select Program M2. For example, M1 and M2 might be two dosages of the same drug; if you chose to administer M1, which requires giving 5 mg of Drug A per day, you could not at the same time implement M2, 10 mg of Drug A." (page 280)		L I

Publication	Definition of "mutually exclusive"	Definition of "independent"	Type of definition
Johannesson, 1996[6]	“Two programmes, A and B, are mutually exclusive if implementing programme A (B) means that programme B (A) cannot also be implemented. If the programmes A and B can both be implemented physically, but implementing A (B) means that the costs and effectiveness of B (A) change, then the programmes should be defined as mutually exclusive. In this case, we have three alternatives: carrying out only A, carrying out only B, or carrying out both A and B. Mutually exclusive programmes can be viewed as programmes for the same population, e.g. two alternative drugs for ulcer patients." (page 135)	"Two programmes, A and B, are defined as independent if the costs and effectiveness of programme A (B) are not affected by whether programme B (A) is implemented or not. The two programmes are viewed as applying to two different populations; an example would be the treatment of ulcer patients and the treatment of arthritis patients…” (page 135)	L I P
Johannesson and O'Conor, 1997[13]	“Two programmes are mutually exclusive if the costs and/or effects of one programme are affected by whether the other programme is implemented or not, e.g. two alternative drug therapies to lower blood pressure.”	“Two programmes are independent if the costs and effects of a programme are not affected by whether the other programme is implemented or not, e.g. a treatment for ulcer and a treatment for cancer.”	I
Karlsson and Johannesson, 1996[8]	"The treatments that are available to one patient group are mutually exclusive, which means that a patient can only receive one of the treatment alternatives"	"The treatment used in one patient group is assumed to be independent of the treatments used in other groups. That is the costs and health effects of a treatment in one patient group are not affected by the treatment alternative chosen in any other patient group"	L I P
Philips 2009[57]	“A distinction must be made between those interventions that are completely **independent –** that is, where the costs and effects of one intervention are not affected by the introduction or otherwise of other interventions – and those that are mutually exclusive **–** that is, where implementing one intervention means that another cannot be implemented, or where the implementation of one intervention results in changes to the costs and effects of another.”		L I
Weinstein, 1996[58]	“Many applications of cost-effectiveness analysis in health care involve comparisons among competing alternatives for the same condition. [...] In such competing choice situations, the basic cost effectiveness paradigm [for independent treatments] no longer applies without modification, because the alternatives (e.g. different drugs for the same condition) are no longer independent. As an obvious example, the benefits of giving two antihypertensive drugs to the same group of patients are not additive. The paradigm needs to be modified to incorporate the possibility of mutually exclusive competing choices for the same condition.”	"The costs and effectiveness of any programme are independent of which other programmes are adopted."	I P

Publication	Definition of "mutually exclusive"	Definition of "independent"	Type of definition
WHO[18]	"Interventions are said to be mutually exclusive if only one alternative can be selected. Mutually exclusive interventions have also been called "competing" or "incompatible" interventions. […] Interventions are said to be independent if choosing one does not prevent the choice of any other intervention." (endnote 3)<br>"Many interventions interact in terms of either costs or effects at the population level. The health impact of undertaking two interventions together is not necessarily additive, nor are the costs of the joint production. To understand whether they are efficient uses of resources independently or in combination requires assessing their costs and health effects independently and in combination. […] Interventions that interact should be evaluated as a group […] The case of mutually exclusive options is similar, i.e. interventions which by definition cannot be implemented simultaneously in the same population. An example is population-based annual and biannual breast cancer screening. These interventions must be evaluated as part of the same set which will ensure that only one of the interventions appears in an optimal mix." (page 21)<br>The term 'mutually exclusive' is used to describe the combinations of treatments, such as DOTS alone, BCG 50% coverage alone and DOTS + BCG 50% coverage (page 21)<br>Discusses "Clusters of interventions that are interrelated either because they interact on either costs or effects or because they are mutually exclusive" (page 25)		L I+

* The second edition[59] (pages 131-2) gave almost identical definitions to the third, while the first edition[60] does not give definitions.
