# Supplementary material for: Decision Making for Healthcare Resource Allocation: Joint v. Separate Decisions on Interacting Interventions
Source: Med Decis Making. 2018 Apr 23;38(4):476–86. doi: 10.1177/0272989X18758018 (PMC5949981; doi:10.1177/0272989X18758018)
Supplement: Appendix_2 [file Appendix_2.pdf]

Appendix 2: Examples of different types of interaction

This appendix describes worked examples illustrating different mechanisms by which interactions can arise. Within each of the examples shown, interactions are calculated as $\mu_0 + \mu_{ab} - \mu_a - \mu_b$ (the outcomes for *0* – outcomes for *a* – outcomes for *b* + outcomes for *ab*).[15,16] Interactions are always calculated on a natural scale, since economic evaluation results must be interpreted on a natural scale (e.g. calculating the ICER as the absolute difference in cost divided by the absolute difference in effects). For simplicity, no discounting is applied in any of the examples, although this would not affect the conclusions.

Unless otherwise stated, all examples are hypothetical and should not be interpreted as reflecting actual clinical practice, efficacy or cost.

Interaction type 1: Direct pharmacological, behavioural or biological mechanisms

The Self Management in OA of the Hand (SMOotH) factorial trial evaluated the impact of hand exercises plus joint protection, hand exercises only, joint protection only and neither treatment.[23]

A within-trial economic evaluation identified a qualitative interaction for QALYs, which meant that hand exercises alone generated the largest number of QALYs, while adding hand exercises to joint protection reduced the number of QALYs.[23] There was also a small synergistic interactions for cost. Although the exact mechanism for this interaction is unclear, it could arise from behavioural mechanisms, such as patients finding it difficult to comply with two interventions at once.

Table A2. Costs and QALYs from the SMOotH trial; the costs and QALYs for each study arm are reproduced with permission from Table 5 of Oppong, et al 2015 (Rheumatology 54(5): 876-883)

	Cost	QALYs	NMB*
Neither treatment	£58.46	0.662	£13,182
Hand exercises only	£64.51	0.681	£13,555
Joint protection only	£92.12	0.659	£13,088
Joint protection plus hand exercises	£112.38	0.658	£13,048
Interaction	£14.21	-0.020	-£414

* At a £20,000/QALY ceiling ratio.

Interaction type 2a: Multiplicative effects on the odds of clinical events

The rate of fatal and non-fatal cardiovascular disease (CVD) events was calculated from data presented in a published factorial trial evaluating antihypertensive (AH, candesartan/hydrochlorothiazide) and statin (rosuvasatin) against their respective placebos (Table A3).[61] The second co-primary endpoint from the trial (number of CVD events) was analysed using Cox proportional hazards and the study observed no statistically significant interactions between CH and rosuvastatin.[61]

However, an interaction arises for absolute event rates as statins and antihypertensives both decrease the rate of CVD events by a certain proportion, which tends to give no interaction on a logarithmic scale, but an antagonistic interaction for the number of events and the rate of events. We calculated a simple estimate of lifetime cost-effectiveness by applying published estimates of the cost and utility decrements associated with CVD events[62,63] to the event rates observed in this study using the assumptions shown in Table A4). This demonstrates that the antagonistic interaction for CVD event rates leads to an antagonistic

interaction for QALYs and synergistic effects on costs (Table A3). This analysis is intended to illustrate the mechanisms by which interactions may arise, but makes a number of assumptions and is not intended to indicate the costs, QALYs or cost-effectiveness of either intervention in routine clinical practice.

Table A3. Outcomes

	**AH + statin**	**Statin only**	**AH only**	**No treatment**	**Inter-action**	**Source**
No. CVD events	136	141	176	187	-	Yusuf et al, Table 2[61]
No. CVD deaths	75	79	80	91	-	Calculated from Yusuf et al, Table 2[61]
No. patients in trial	3180	3181	3176	3168	-	Yusuf et al, Table 2[61]
Mean patient-years of follow-up	4.840	4.856	4.856	4.801		Calculated from Yusuf et al, Figure 1, panel A[61] using life-table method
Rate: total events	0.00884	0.00913	0.01141	0.01229	0.00059	Rate = events/(number of patients*mean follow-up)
Rate: fatal events	0.00487	0.00511	0.00519	0.00598	0.00056	
Rate: non-fatal events	0.00396	0.00401	0.00622	0.00631	0.00004	
Total events/pt over 20 years	0.22091	0.22819	0.28528	0.30737	0.01481	Rate multiplied by 20
Drug cost/patient	£9,207	£5,865	£3,341	£0	£1	Drug cost anticipated over patients' lifetime, allowing for reductions in life expectancy from fatal events
Total costs of events/patient	£4,784	£4,867	£7,206	£7,398	£108	Number of fatal and non-fatal events multiplied by relevant costs
Total cost/patient	£13,991	£10,732	£10,547	£7,398	£110	Drug cost plus event cost
QALYs/patient	18.25	18.19	18.14	17.95	-0.136	Number of fatal and non-fatal events multiplied by relevant QALY losses
NMB/patient	£351,065	£353,133	£352,301	£351,542	-£2,827	At £20,000/QALY ceiling ratio

Table A4. Data inputs

Drug cost/day: Candesartan 16 mg + hydrochlorothiazide 12.5mg	£0.367	UK drug tariff price based on losartan 100mg / hydrochlorothiazide 12.5mg, since candesartan/ hydrochlorothiazide is not yet commercially available[64]
Drug cost/day: Rosuvasatin 10 mg	£0.644	UK tariff price[64]
% events that are MI	18%	Calculated from data in Table 2 in Yusuf et al[61]
% events that are stroke	26%	Calculated from data in Table 2 in Yusuf et al[61]
% events that are IHD	56%	Calculated from data in Table 2 in Yusuf et al[61]
% events that were fatal	51%	Calculated from data in Table 2 in Yusuf et al[61]
Life expectancy in the absence of events	20	Assumption
Mean years elapsed before each event	10	Assumption
EQ-5D utility in the absence of events	0.78	UK average EQ-5D utility for 65-74-year-olds[65]
QALY loss from fatal events	7.8	Mean EQ-5D utility multiplied by years elapsed between event and life expectancy
QALY loss from non-fatal events	0.491	Calculated from the event costs and utility decrements for 70-year-old men calculated by Alva et al,[62,63] based on the case fatality and event breakdown above
Cost of fatal events	£4,981	
Cost of non-fatal events	£35,849	

If we were to make a joint decision on statins and antihypertensives allowing for this interaction, we would evaluate no treatment, antihypertensive only, statin only and statin+antihypertensive incrementally as four mutually exclusive treatment combinations (Table A5). Based on this analysis, we would (at a £20,000/QALY ceiling ratio) adopt statin monotherapy (costing £13,541/QALY versus no treatment), since antihypertensive monotherapy is weakly dominated and combination therapy costs £54,760/QALY versus statin alone.

Table A5. Incremental outcomes per patient treating the options as interdependent or mutually exclusive

	Incr Cost	Incr QALYs	ICER
Antihypertensive only vs. no treatment	£3,149	0.1954	£16,119 (weakly dominated)
Statin only vs. antihypertensive	£3,334	0.2463	£13,541
Statin + antihypertensive vs. statin	£3,259	0.0595	£54,760

Making separate decisions on the two treatments requires some assumption about the proportion of patients who will receive the concomitant treatment. In practice, this may be done implicitly or inconsistently: e.g. by ignoring the drug cost for the concomitant treatment, but basing clinical effectiveness estimates on the available studies, which may or may not include patients receiving concomitant therapy.

If we assume that no patients have concomitant treatment (i.e. assume that no patients have antihypertensives when we evaluate statin vs. no statin), we would obtain the same ICERs as we would if we evaluated the different combinations incrementally in a joint decision, but adopt both statins and antihypertensives, since both have ICERs <£20,000/QALY. If we assumed that 50% of people would receive concomitant treatment, we would adopt statin monotherapy (the same conclusion as a joint decision). Conversely, if we assumed that all patients receive the concomitant treatment, we would adopt neither statins nor antihypertensives.

Table A6. Incremental outcomes per patient treating the options as independent

	Incr Cost	Incr QALYs	ICER
Assuming that no patients received concomitant treatment			
Antihypertensive vs. no antihypertensive	£3,149	0.1954	£16,119
Statin vs. no statin	£3,334	0.2463	£13,541
Assuming that 50% of patients received concomitant treatment			
Antihypertensive vs. no antihypertensive	£3,204	0.1274	£25,141
Statin vs. no statin	£3,389	0.1783	£19,006
Assuming that all patients received the concomitant treatment			
Antihypertensive vs. no antihypertensive	£3,259	0.0595	£54,760
Statin vs. no statin	£3,444	0.1104	£31,198

Sequencing the appraisals, such that we assess one intervention first (assuming no concomitant treatment) and then assess the second intervention afterwards, conditional on having adopted the most cost-effective treatment in the first decision will not necessarily give the correct conclusion: even if we evaluate cost-effectiveness for patients of different cardiovascular risk. For example, if we were to assess antihypertensives first (assuming that no patients have statins), we would adopt antihypertensives in the first stage on the grounds that they cost £16,119/QALY gained. Given that we have adopted antihypertensives, we would not then adopt statin+antihypertensive, since it costs £31,198/QALY versus antihypertensives alone. However, the optimal treatment (statin monotherapy) would never be assessed and would therefore never be adopted if antihypertensives were evaluated first. This approach may reduce the net loss of NMB from ignoring the interaction by avoiding inappropriate use of combination therapy, but nonetheless fails to exclude dominated options and may result in adoption of the first treatment evaluated (or no treatment at all) when the other treatment would have been better value for money.

Interaction type 2b: Multiplicative effects between quality and length of life

Knee replacement doubles patients' utility for all remaining life-years, while smoking cessation interventions (if successful) increase life expectancy by 50%. As a result, there is a large synergistic interaction for QALYs, but additive effects on cost (since both interventions are assumed to be one-off). The actual interaction could be substantially larger than this if stopping smoking before knee replacement surgery reduced operative mortality and morbidity. We can eliminate the interaction by stratifying by life expectancy.

Table A7. Data inputs

Life expectancy: 70-year-old smoker	8
Life expectancy: 70-year-old ex-smoker	12
Utility without knee replacement	0.3
Utility with knee replacement	0.6
Cost of knee replacement	£5,000
Lifetime cost of smoking-related diseases - smoker	£2,000
Lifetime cost of smoking-related diseases - ex-smoker	£1,000
Cost of smoking cessation intervention	£50
Effectiveness of smoking intervention: % of smokers who stop	20%
Population	60,000

Table A8. Outcomes for each treatment combination

	**Population Cost†**	**Population QALYs‡**	**Population NMB§**
No treatment	£120,000,000	144,000	£2,760,000,000
Smoking cessation only	£111,000,000	158,400	£3,057,000,000
Knee replacement only	£420,000,000	288,000	£5,340,000,000
Knee replacement + smoking cessation	£411,000,000	316,800	£5,925,000,000
Interaction	£0	14,400	£288,000,000

† Cost = intervention cost + cost of smoking-related diseases for smokers*proportion not quitting + cost of smoking-related diseases for ex-smokers*proportion quitting, all multiplied by population size
‡ QALYs = Population*relevant utility*(life expectancy for smokers*proportion not quitting + life expectancy for ex-smokers*proportion quitting)
§ At a £20,000/QALY ceiling ratio.

Interaction type 2c: Multiplicative effects on drug cost

Ranibizumab and bevacizumab can both be used to treat age-related macular degeneration and can each be used monthly or as needed. The four dosing regimens were compared in an economic evaluation conducted alongside a factorial trial.[31] An interaction for cost arises because the cost of ranibizumab (£742.17/dose) is more than 15 times higher than the cost of bevacizumab (£49/dose), regardless of the dosing frequency, which means that increasing dosing frequency increases costs more for the more costly drug (ranibizumab). The interaction cannot be eliminated during the analysis: it is essential to make a joint decision on dosing frequency and which drug to use.

Table A9. Costs and outcomes for each treatment over two years.[31] Reproduced with permission from Table 5 of Dakin, et al 2015 (BMJ Open 2014;4(7):e005094)

	Total cost/patient	QALYs/patient	NMB*
As-needed bevacizumab	£3,002	1.584	£28,683
Monthly bevacizumab	£3,601	1.604	£28,480
As-needed ranibizumab	£11,500	1.582	£20,142
Monthly ranibizumab	£18,590	1.608	£13,576
Interaction	£6,491	0.006	-£6,363

* At a £20,000/QALY ceiling ratio.

Interaction type 2d: Multiplicative effects between immediate mortality and remaining life expectancy

Measles mumps and rubella (MMR) vaccination reduces the risk of infant mortality but is (in this simplified example) assumed to have no major impact on subsequent HRQoL or life expectancy. However, the benefits of saving a life in childhood depend on the number of QALYs that will be accrued in the remaining life expectancy. These QALYs are assumed to be substantially increased by hypothetical interventions that completely prevent cardiovascular disease and therefore greatly increases life expectancy. There is therefore a large synergistic interaction for QALYs. In practice, such effects are likely to introduce interactions for cost due to the impact of future costs, although these are omitted from this example.

Table A10. Data inputs

Odds of mortality from MMR by age 5 without vaccination	2.00%
Odds ratio of mortality from MMR by age 5 with vaccination	0.05
Quality-adjusted life expectancy at age 5 given current treatment	60
Quality-adjusted life expectancy at age 5 with a new treatment that prevents all cardiovascular disease	90
Cost of MMR vaccine	£10
Lifetime net cost of treatment preventing cardiovascular disease (drug cost minus savings from disease prevented)	£200,000
Population (babies per year)	600,000

Table A11. Outcomes for each treatment combination

	Population Cost†	Population QALYs‡	Population NMB§
No treatment	£0	35,294,118	£705,882,352,941
MMR vaccine only	£6,000,000	35,964,036	£719,274,719,281
CVD prevention only	£120,000,000,000	52,941,176	£938,823,529,412
MMR + CVD prevention	£120,006,000,000	53,946,054	£958,915,078,921
interaction	£0	334,959.2	£6,699,183,170

† Cost = intervention cost multiplied by population size

‡ QALYs = (odds of MMR mortality/(1+ odds of MMR mortality))*quality-adjusted life expectancy*population.

§ At a £20,000/QALY ceiling ratio.

Within this example, it is essential to make a plausible assumption about the subsequent life expectancy when evaluating the cost-effectiveness of MMR. However, the only impact of the MMR vaccine on the cardiovascular intervention is to increase the number of patients who can benefit from it. An evaluation looking at patients who have already survived to adulthood would not be dependent on whether or not MMR is given.

Interaction type 3a: Diminishing marginal effects on utility

This interaction arises directly from the UK EQ-5D-3L time trade-off tariff.[66] Either of the two painkillers will improve patients' response level on the pain domain by 1 level. Given individually, they can improve pain from extreme to moderate, but given together they can eliminate pain altogether. As result, there is an antagonistic interaction for QALYs, since the coefficients for level 3 pain are more than twice as high as those for level 2 pain, in addition to level 3 pain attracting the N3 term.[66] Effects on costs are additive. A joint decision is needed to allow for the interaction. Similar trends would be seen on any other domain.

Table A12. Data inputs

Painkiller 1 cost/4 months	£600
Painkiller 2 cost/4 months	£1,600
EQ-5D utility with no drug (11232)	0.157
EQ-5D utility with painkiller 1 (112**2**2)	0.689
EQ-5D utility with painkiller 2 (112**2**2)	0.689
EQ-5D utility with 2 painkillers (112**1**2)	0.812
Population	30,000

Table A13. Outcomes for each treatment combination

	Population Cost†	Population QALYs‡	Population NMB§
No treatment	£0	1,570	£31,400,000
Painkiller 1 only	£18,000,000	6,890	£119,800,000
Painkiller 2 only	£48,000,000	6,890	£89,800,000
Painkillers 1 & 2	£66,000,000	8,120	£96,400,000
interaction	£0	-4,090	-£81,800,000

† Cost = intervention cost multiplied by population size
‡ QALYs = Population*relevant utility*0.33 years
§ At a £20,000/QALY ceiling ratio.

Interaction type 3b: Increasing marginal effects on utility

This interaction arises directly from the UK EQ-5D-3L time trade-off tariff.[66] The painkiller reduces patients' pain from extreme to moderate (i.e. from level 3 to level 2 on EQ-5D), while the antidepressant has the same effect on the anxiety/depression domain. As result, there is a large synergistic interaction on utilities and QALYs due to the EQ-5D tariff, which subtracts 0.269 from utility for any patients with level 3 on any domain:[66] both anxiety/depression and pain must be improved to above level 3 to remove the N3 term.

Table A14. Data inputs

Painkiller cost/4 months	£900
Antidepressant cost/4 months	£250
EQ-5D utility with no drug (11233)	-0.008
EQ-5D utility with painkiller (112**2**3)	0.255
EQ-5D utility with antidepressant (1123**2**)	0.157
EQ-5D utility with painkiller & anxiety and depression (112**22**)	0.689
Population	20000

Table A15. Outcomes for each treatment combination

	Population Cost†	Population QALYs‡	Population NMB§
No treatment	£0	-53	-£1,066,667
Antidepressant only	£5,000,000	1,047	£15,933,333
Painkiller only	£18,000,000	1,700	£16,000,000
Painkiller + antidepressant	£23,000,000	4,593	£68,866,667
interaction	£0	1,793	£35,866,667

† Cost = intervention cost multiplied by population size
‡ QALYs = Population*relevant utility*0.33 years
§ At a £20,000/QALY ceiling ratio.

A joint decision is needed to allow for the interaction. Similar effects would also be seen for other domains and for combinations of interventions that improve patients to perfect health. Even larger interactions would be seen if improved pain relief also improved anxiety/depression or vice versa.

Interaction type 3c: Ceiling effects for HRQoL

Treatments A and B are evaluated in a 10-year trial. The treatments have no effect on length of life, but in the absence of any ceiling effect, treatment A would increase utility by 0.15 and B would increase utility by 0.2, with no interaction. The treatments have additive effects on cost. However, since utility cannot be increased above 1, patients cannot accrue more than 10 QALYs in this 10-year trial (no discounting is applied for simplicity, although if it were applied it would simply decrease QALYs by the same proportion in all patients).

In this example, the ceiling effect does not come into play for the average patient, since the simple effects would suggest that the mean utility for the average patient receiving treatment *ab* is 0.95 (0.6+0.15+0.2). However, when we take account of the distribution of QALYs by randomly sampling from normal distributions with SD equalling 0.2, the proportion of patients accruing 10 QALYs (the maximum) is substantially higher for the *ab* group, introducing a large antagonistic interaction for QALYs. Although the interaction for cost (and the interaction for QALYs in the absence of the ceiling effect) would be zero if infinite numbers of patients were sampled, the use of Monte Carlo simulation means that there is a small interaction for cost within this sample of 500 patients. Similar trends would be seen for life-extending treatments within a trial of fixed duration, even if the effect of treatment on mortality were not additive.

Table A16. Data inputs

Time horizon of the study (years)	10
Maximum utility	1
Mean utility no treatment	0.6
Increase in utility from treatment A	0.15
Increase in utility from treatment B	0.2
SD around utility	0.2
Cost of A	£2,000
Cost of B	£3,000
Cost without treatment	£1,000
SD around cost without treatment	£300
Population	10,000

Table A17. Outcomes for each treatment combination

	**Population Cost†**	**Population QALYs‡**			**Population NMB§**
		**If there were no ceiling effect**	**% patients with 10 QALYs**	**With ceiling effect**	**With ceiling effect**
No treatment	£9,855,731	59,837	3%	59,646	£1,183,070,245
A only	£30,050,502	75,348	9%	74,395	£1,457,858,878
B only	£40,022,721	79,724	15%	78,179	£1,523,555,682
A and B	£60,093,652	95,507	40%	89,603	£1,731,971,226
Interaction	-£123,839	273	19%	-3,325	-£66,373,089

† Cost was estimated for each of 500 simulated patients by sampling values from a gamma distribution representing cost without treatment with mean £1,000 and SD £300 (alpha = 11.11, beta = 90). Treatment costs were added to the cost without treatment and were assumed to be fixed. Population cost was calculated by averaging across the 500 trial participants and multiplying the average by the population size.
‡ QALYs without a ceiling effect were calculated for each of 500 simulated patients by sampling values from normal distribution with a mean equal to the group mean for that treatment arm and a SD of 0.2. Population QALYs were calculated by averaging over the 500 trial participants and multiplying the average by the population size.
§ At a £20,000/QALY ceiling ratio.

Interaction type 4a: Earlier intervention affects costs and benefits of later intervention and vice versa)

Treating the patients diagnosed with cervical cancer with the new treatment rather than the comparator treatment increases costs and improves quality-adjusted survival for patients at all cancer stages and therefore increases the cost and reduces the benefits of screening.[47] Conversely, screening patients for cervical cancer will increase the chance that cancers are detected at an early stage (e.g. stage I); total costs and quality adjusted survival vary depending on the stage of cancer at which patients are diagnosed. Additionally, the incremental costs and incremental QALYs associated with the new treatment compared with its comparator also vary with stage.

As a result of this, if we consider cervical cancer as a whole (rather than making separate decisions for different stages), we get an antagonistic interaction for costs and a synergistic interaction for QALYs.

Table A18. Data inputs

No. women in population (millions)	20			
Cost of screening (per woman screened)	£100			
Prevalence of cervical cancer	2%			
QALYs if don't develop cancer	40			
Data inputs by cancer stage				
Cancer stage	I	II	III	IV
Probability diagnosed at this stage if not screened	50%	30%	15%	5%
Probability diagnosed at this stage if screened	79%	15.0%	5.0%	1.0%
QALYs if diagnosed at this stage: comparator treatment	20	10	5	1
QALYs if diagnosed at this stage: new treatment	30	15	7.5	1.5
Cost if diagnosed at this stage: comparator treatment	£5,000	£10,000	£15,000	£30,000
Cost if diagnosed at this stage: new treatment	£7,500	£15,000	£22,500	£45,000

Table A19. Outcomes and costs by stage for each treatment combination

Cost-effectiveness of treatment	**Cost per million in population**			**QALYs per million in population**			**ICER: New Tx vs. comparator**
	**New Tx†**	**Compa-rator†**	**Δ**	**New Tx‡**	**Compa-rator‡**	**Δ**	
Stage I - without screening	£1,500	£1,000	£500	6.00	4.00	2.00	£250
Stage II - without screening	£1,800	£1,200	£600	1.80	1.20	0.60	£1,000
Stage III - without screening	£1,350	£900	£450	0.45	0.30	0.15	£3,000
Stage IV - without screening	£900	£600	£300	0.03	0.02	0.01	£30,000
All stages combined - without screening, but including QALYs for patients without cervical cancer	£5,550	£3,700	£1,850	792.28	789.52	2.76	£670
Stage I - with screening	£2,370	£1,580	£790	9.48	6.32	3.16	£250
Stage II - with screening	£900	£600	£300	0.90	0.60	0.30	£1,000
Stage III - with screening	£450	£300	£150	0.15	0.10	0.05	£3,000
Stage IV - with screening	£180	£120	£60	0.01	0.00	0.00	£30,000
All stages combined - with screening and costs and QALYs for patients without cervical cancer	£5,900	£4,600	£1,300	794.54	791.02	3.51	£370

† Cost = population * prevalence of cervical cancer * proportion of patients diagnosed at this stage * cost if diagnosed at this stage. The all stages combined cost with screening includes the cost of screening (equal to population*cost of screening).

‡ QALYs = population * prevalence of cervical cancer * proportion of patients diagnosed at this stage * QALYs if diagnosed at this stage.

Table A20. Outcomes for each treatment combination

	**Population Cost†**	**Population QALYs‡**	**Population NMB§**
No cervical screening and old cervical cancer treatment	£3,700,000,000	789,520,000	£15,786,700,000,000
Cervical screening and old cervical cancer treatment	£4,600,000,000	791,024,000	£15,815,880,000,000
No cervical screening and new cervical cancer treatment	£5,550,000,000	792,280,000	£15,840,050,000,000
Cervical screening and new cervical cancer treatment	£5,900,000,000	794,536,000	£15,884,820,000,000
Interaction	-£550,000,000	752,000	£15,590,000,000

† Costs taken from Table A19.
‡ QALYs taken from Table A19.
§ At a £20,000/QALY ceiling ratio.

However, providing that screening doesn't affect the costs and QALYs of patients within each cancer stage, the cost-effectiveness of treating cervical cancer will be independent of screening if we make separate decisions about treatment for each cancer stage. Once we have made the decision about the best cancer treatment, we can then make a decision on the cost-effectiveness of screening that is contingent on patients receiving the most cost-effective treatment strategy.

Although this example is based on hypothetical data, the interaction between treatment and screening has also been demonstrated empirically, without stratifying patients by cancer stage.[47]

Interaction type 4b: Interactions between diseases

Drug A decreases the risk of developing heart failure, but has no direct effect on the risk of stroke. Drug B decreases the risk of stroke, but has no direct effect on the risk of heart failure.

However, patients with heart failure are at increased risk of stroke and the two events have multiplicative effects on cost and HRQoL, such that the HRQoL impact of heart failure and stroke is less than that of the two diseases separately, while the additional cost of managing patients with both heart failure and stroke is greater than the effect of each disease separately.

The interaction between these two diseases was modelled using a Markov model, to estimate the number of patients in each of the five disease states (no disease, stroke, heart failure, stroke + heart failure and dead). 25 annual cycles were modelled with a half cycle correction. Development of stroke and heart failure were assumed to be permanent.

Table A21. Data inputs

Annual Odds of heart failure	0.03
Odds ratio for heart failure Drug A	0.4
Annual Odds of stroke (without heart failure)	0.05
Odds ratio for stroke Drug B	0.5
Odds ratio for stroke: in patients with heart failure vs. those without	2
Annual cost with neither stroke nor heart failure	1,000
Annual cost of stroke alone	£15,000
Annual cost of heart failure alone	£4,000
Annual cost of stroke and heart failure	£19,000
Utility with neither stroke nor heart failure	0.8
Utility with stroke alone	0.32
Utility with heart failure alone	0.56

Utility with stroke and heart failure	0.08
Annual odds of death without either heart failure or stroke	0.02
Odds ratio of death after developing heart failure	4
Odds ratio of death after stroke	7
Drug cost/year: A (heart failure)	£200
Drug cost/year: B (stroke)	£300
Population	500,000

Table A22. Outcomes for each treatment combination

	Population Cost†	Population QALYs‡	Population NMB§
No treatment	28,703,311,049	4,410,346	£59,503,618,166
Drug A (reduces risk of heart failure)	32,132,300,059	4,864,927	£65,166,240,290
Drug B (reduces risk of stroke)	25,237,151,910	5,300,440	£80,771,641,099
Drug A + Drug B	26,267,367,316	5,854,271	£90,818,057,439
Interaction	-£2,398,773,604	99,251	£4,383,794,216

† Annual cost = sum product of the annual costs of each disease state and the number of patients each disease state. Drug A was assumed to be given to living patients who had not yet developed heart failure, while drug B was assumed to be given to living patients who had not yet had a stroke. Annual costs were summed over all years, with a half cycle correction applied to cycles zero and 25.
‡ QALYs = sum product of the utility of each disease state and the number of patients in each disease state. Annual QALYs were summed over all years, with a half cycle correction applied to cycles zero and 25.
§ At a £20,000/QALY ceiling ratio.

Interaction type 4c: Effect of comorbid conditions on treatment costs

The cost of conducting knee replacement surgery is substantially higher for morbidly obese patients, since specialised beds, instruments, etc. are required. For the population of people with morbid obesity and knee osteoarthritis, there is therefore a mixed interaction for cost between knee replacement surgery and interventions for obesity (e.g. bariatric surgery) that are conducted before knee surgery.

Table A23. Data inputs

Cost of knee replacement: non-morbidly obese patients	£6,000
Cost of knee replacement: morbidly-obese patients	£10,000
Cost of obesity surgery	£8,000
Utility with morbid obesity and knee osteoarthritis	0.2
Utility improvement from obesity surgery	0.15
Utility improvement from knee replacement	0.3
Life expectancy: non-morbidly obese patients (years)	20
Life expectancy: morbidly obese patients (years)	20
Proportion of patients ceasing to be morbidly obese after obesity surgery	60%
Additional annual cost of managing morbidly obese patient compared with non-morbidly obese patient	£1,500
Population: number of patients with knee osteoarthritis and morbid obesity	20,000

Table A24. Outcomes for each treatment combination

	Population Cost†	Population QALYs‡	Population NMB§
No knee replacement and no obesity surgery	£600,000,000	80,000	£1,000,000,000
Obesity surgery only	£400,000,000	116,000	£1,920,000,000
Knee replacement only	£800,000,000	200,000	£3,200,000,000
Obesity surgery and knee replacement	£552,000,000	236,000	£4,168,000,000
Interaction	-£48,000,000	0	£48,000,000

† Cost = cost of any of obesity surgery, plus the proportion of patients ceasing to be morbidly obese multiplied by the cost of knee replacement, plus the proportion of patients remaining morbidly obese multiplied by the cost of knee replacement and the lifetime cost of managing obesity (all multiplied by the population size).
‡ QALYs = proportion of patients ceasing to be morbidly obese multiplied by life expectancy for non-morbidly obese patients multiplied by the HRQoL with/without knee replacement, plus proportion of patients to be morbidly obese multiplied by life expectancy for morbidly obese patients multiplied by the HRQoL with/without knee replacement (all multiplied by the population size).
§ At a £20,000/QALY ceiling ratio.

Interaction type 4d: Impact of future costs

Future costs introduce an interaction between a smoking cessation intervention at age 65 and the introduction of unlimited free nursing care. Introducing free nursing care is assumed to increase the cost of care by £5,000 in every year of life. Patients who have a longer life expectancy will therefore accrue a larger cost from the free nursing care. Since the smoking cessation intervention increases life expectancy, those patients accrue higher costs in the years of life gained and (if these future costs are included in the analysis), the incremental cost of smoking cessation is higher with free nursing care than without. In this example, there is also a small synergistic interaction for QALYs due to the same effect observed in example B.

Table A25. Data inputs

Incremental cost per year of life of unlimited free nursing care to NHS	£5,000
Utility with unlimited free nursing care	0.75
Utility without unlimited free nursing care	0.7
Lifetime cost of smoking-related diseases - smoker	£2,000
Lifetime cost of smoking-related diseases - ex-smoker	£1,000
Cost of smoking cessation intervention	£50
Effectiveness of smoking intervention: % of smokers who stop	20%
Life expectancy smoker	18
Life expectancy ex-smoker	22
Population	3,000,000

Table A26. Outcomes for each treatment combination

	Population Cost†	Population QALYs‡	Population NMB§
No treatment	£6,000,000,000	37,800,000	£750,000,000,000
Smoking cessation intervention only	£5,550,000,000	39,480,000	£784,050,000,000
Free nursing care only	£270,000,000,000	40,500,000	£540,000,000,000
Smoking cessation and free nursing care	£287,550,000,000	42,300,000	£558,450,000,000
Interaction	£18,000,000,000	120,000	-£15,600,000,000

† Cost = cost of smoking cessation intervention (if relevant) + annual cost of nursing care*weighted average life expectancy + weighted average cost of smoking-related diseases, all multiplied by population size. Life expectancy and the cost of smoking-related diseases were averaged over the proportion of people who quit smoking and the proportion who do not.
‡ QALYs = Population*relevant utility*(life expectancy for smokers*proportion not quitting + life expectancy for ex-smokers*proportion quitting).
§ At a £20,000/QALY ceiling ratio.

Interaction type 5: Interactions arising from shared capital expenditure

A hospital currently has no MRI machine. They could introduce facilities for MRI scans of patients with head injuries and/or they could introduce facilities for MRI scans of patients with suspected cancer. Within this simplified example, the MRI machine has capacity for at least 5,000 patients and would not be used for any other patients, nor would patients be referred elsewhere. However, similar principles will apply even if these simplifications were relaxed.

Because the machine can be used for both services, but only needs to be purchased once, the interaction for cost is exactly equal to the cost of the MRI machine. By contrast, in this example there is no interaction for QALYs.

Table A27. Data inputs

Cost of purchasing MRI machine	£500,000
Variable costs per scan	£50
QALYs with head injury without MRI scan	30
QALYs with suspected cancer without MRI scan	15
QALY gained from MRI scan for head injuries	1
QALY gained from MRI scan for suspected cancer	0.5
Incremental cost of scanning for head injuries excluding cost of scan	-£50
Incremental cost of scanning for suspected cancer excluding cost of scan	£300
Number of scans for head injuries per MRI machine	3,000
Number of scans for cancer per MRI machine	2,000

Table A28. Outcomes for each treatment combination

	Population Cost†	Population QALYs‡	Population NMB§
No MRI	£0	120,000	£2,400,000,000
MRI for head injuries only	£500,000	123,000	£2,459,500,000
MRI for cancer only	£1,200,000	120,008	£2,398,950,000
MRI for both head injuries and cancer	£1,200,000	123,008	£2,458,950,000
Interaction	-£500,000	0	£500,000

† Cost = cost of MRI machine (if relevant) + number of scans for head injuries*(variable cost per MRI scan + incremental cost of scanning for head injuries) + number of scans for cancer*(variable cost per MRI scan + incremental cost of scanning for cancer).
‡ QALYs = Number of head injuries*number of QALYs accrued with head injuries + number of suspected cancer cases*number of QALYs for patients with suspected cancer
§ At a £20,000/QALY ceiling ratio.

No interaction (additive effects)

Since treatments for ovarian cancer and benign prostatic hypertrophy (BPH) are given to different, non-overlapping patient populations with different diseases managed by different staff in different healthcare facilities, they are completely independent and have additive effects on costs and QALYs. It is therefore reasonable to make independent decisions on the two treatments.

Table A29. Data inputs

	Total cost/patient	Total QALYs/patient
Ovarian cancer treatment	£7,500	30
Ovarian cancer no treatment	£5,000	20
BPH treatment	£15,000	29
BPH no treatment	£500	28.5
Number of cases of ovarian cancer	5,000	
Number of cases of BPH	8,000	

Table A30. Outcomes for each treatment combination

	Population Cost†	Population QALYs‡	Population NB§
No treatment	£29,000,000	328,000	£6,531,000,000
Ovarian cancer treatment only	£41,500,000	378,000	£7,518,500,000
BPH treatment only	£145,000,000	332,000	£6,495,000,000
Ovarian cancer treatment + BPH treatment	£157,500,000	382,000	£7,482,500,000
Interaction	£0	0	£0

† Cost = Cost of ovarian cancer*number of cases of ovarian cancer + cost of BPH*number of BPH cases
‡ QALYs = QALYs with ovarian cancer*number of ovarian cancer cases + QALYs with BPH*number of BPH cases
§ At a £20,000/QALY ceiling ratio.

Because there is no interaction for either costs or QALYs, incremental costs, incremental QALYs and ICERs are the same regardless of whether we make independent decisions (treating the options as independent, Table A31) or as a joint decision (treating the options as mutually exclusive, Table A32).

Table A31. Incremental outcomes per thousand population treating the options as independent

	Incr Cost	Incr QALYs	ICER
Ovarian cancer treatment	£12,500	50.00	£250
BPH treatment	£116,000	4.00	£29,000

Table A32. Incremental outcomes per thousand population treating the options as interdependent or mutually exclusive

	Incr Cost	Incr QALYs	ICER
Ovarian cancer treatment only vs. no treatment	£12,500	50.00	£250
BPH treatment only vs. ovarian cancer treatment only	£103,500	-46.00	Ex dominated
Ovarian cancer treatment + BPH treatment vs. ovarian cancer treatment only	£116,000	4.00	£29,000
