# Supplementary material for: Decision Making for Healthcare Resource Allocation: Joint v. Separate Decisions on Interacting Interventions
Source: Med Decis Making. 2018 Apr 23;38(4):476–86. doi: 10.1177/0272989X18758018 (PMC5949981; doi:10.1177/0272989X18758018)
Supplement: Appendix_3 [file Appendix_3.pdf]

Appendix 3: Table for identification and assessment of interactions

Table A33. Blank table for identification and assessment of interactions

		**Is it physically possible to implement both interventions simultaneously?**	**Are there reasons to expect the interventions to interact?**	**Is the interaction likely to be large enough to change the conclusions?**	**Are there ways to mitigate the interaction in the analysis?**	**Is a joint assessment necessary?**
**Primary population of interest:**						
**Intervention:**						
**What interventions are/could be given in the same pathway?**	1.					
	2.					
	3.					
	4.					
	5.					
	6.					
	7.					
	8.					
	9.					
	10.					

Table A34. Worked example of the table for identification and assessment of interactions: Atorvastatin for primary prevention of CVD. Prepared based on the information reported in the NICE guideline for lipid modification[41]

		**Is it physically possible to implement both interventions simultaneously?**	**Are there reasons to expect the interventions to interact?**	**Is the interaction likely to be large enough to change the conclusions?**	**Are there ways to mitigate the interaction in the analysis?**	**Is a joint assessment necessary?**
**Primary population of interest:**	Patients aged >40 years without diabetes with >5% 10-year risk of CVD					
**Intervention:**	Atorvastatin					
**What interventions are/could be given in the same pathway?**	1. Other statins	Contraindicated	N/A	N/A	N/A	Yes*
	2. Ezetimibe	Yes	Yes: proportional effect on CVD events as both target cholesterol	Potentially†	Unlikely	Yes†
	3. Fibrates	Yes	Potentially, if both had proportional effects on CVD events	Potentially (unless there was evidence that fibrates would not be cost-effective)	Evaluate fibrates first and exclude from consideration if they are very poor value for money	Potentially (if fibrates could be cost-effective)†
	4. Nicotinic acid, omega-3, foods enriched with phytosterols, bile acid sequestrants	Yes	Potentially, if both had proportional effects on CVD events	No: little/no evidence that the treatments are effective	N/A	No
	5. Aspirin	Yes	Yes: proportional effects on CVD events	Potentially†	No	Yes†
	6. Antihypertensives	Yes	Yes: proportional effects on CVD events	Potentially†	No	Yes*†
	7. Diet modification	Yes	Yes: proportional effects on CVD events. May influence compliance	Possibly: lifestyle changes may be cost-effective regardless of statins†	Evaluate lifestyle changes before statins and (if these are highly cost-effective), evaluate statins assuming these are adopted	Potentially (if lifestyle changes are not highly cost-effective)†
	8. Exercise interventions	Yes				
	9. Smoking cessation interventions	Yes				
	10. Interventions for managing acute CVD events	Yes	No	N/A	N/A	No

		Is it physically possible to implement both interventions simultaneously?	Are there reasons to expect the interventions to interact?	Is the interaction likely to be large enough to change the conclusions?	Are there ways to mitigate the interaction in the analysis?	Is a joint assessment necessary?
	11. Drug therapy for secondary prevention	Yes	No	N/A	N/A	No
	12. Interventions for other conditions that extend length of life	Yes	Yes: Both interventions likely to have proportional effects on mortality	No: only likely to affect a subset of patients	No	No
	13. Interventions for other conditions that improve HRQoL	Yes	Yes: By extending length of life, statins will increase benefits of the other intervention. CVD events and comorbid conditions may have non-additive effects on HRQoL.	No: likely to only affect a subset of patients	No	No
	14. Interventions to improve compliance with statins	Yes, although there is no point in compliance intervention without statin treatment	Yes: Compliance interventions likely to produce proportional increase costs and efficacy of statins, but have no effect in the absence of statins	Potentially†	No	Yes†
	15. CVD risk predictors	Yes	Potentially: affects definition of patient population	Unknown†	Select CVD risk predictor before assessing statins	Potentially†

* This drug class includes a large number of different treatments. The decision problem could be greatly simplified by first assessing the cost-effectiveness of different statins against other statins and no treatment and excluding dominated options from further analysis. Similarly, different combinations of antihypertensives could be evaluated against one another and no treatment to exclude dominated options.

† Whether the interaction is large enough to affect the conclusions and whether it is therefore necessary to conduct a joint assessment may depend on cardiovascular risk. In this situation, we examine a relatively low-risk group, where it is assumed that the cost-effectiveness of statins and other interventions may be affected by interactions. However, in high risk patients, interactions may be less likely to affect the conclusions as statins and other treatments may be cost-effective regardless of the assumptions made.
